# Sustainability of Rainwater Harvesting System in terms of Water Quality

**DOI:** 10.1155/2014/721357

**Published:** 2014-02-18

**Authors:** Sadia Rahman, M. T. R. Khan, Shatirah Akib, Nazli Bin Che Din, S. K. Biswas, S. M. Shirazi

**Affiliations:** ^1^Department of Civil Engineering, Faculty of Engineering, University of Malaya, 50603 Kuala Lumpur, Malaysia; ^2^Department of Architecture, Faculty of Built Environment, University of Malaya, 50603 Kuala Lumpur, Malaysia; ^3^Department of Civil Engineering, Bangladesh University of Engineering & Technology, Dhaka 1000, Bangladesh; ^4^Institute of Environmental and Water Resources Management (IPASA), Faculty of Engineering, Universiti Teknologi Malaysia, 81310 Johor, Malaysia

## Abstract

Water is considered an everlasting free source that can be acquired naturally. Demand for processed supply water is growing higher due to an increasing population. Sustainable use of water could maintain a balance between its demand and supply. Rainwater harvesting (RWH) is the most traditional and sustainable method, which could be easily used for potable and nonpotable purposes both in residential and commercial buildings. This could reduce the pressure on processed supply water which enhances the green living. This paper ensures the sustainability of this system through assessing several water-quality parameters of collected rainwater with respect to allowable limits. A number of parameters were included in the analysis: pH, fecal coliform, total coliform, total dissolved solids, turbidity, NH_3_–N, lead, BOD_5_, and so forth. The study reveals that the overall quality of water is quite satisfactory as per Bangladesh standards. RWH system offers sufficient amount of water and energy savings through lower consumption. Moreover, considering the cost for installation and maintenance expenses, the system is effective and economical.

## 1. Introduction

**Figure 1:**
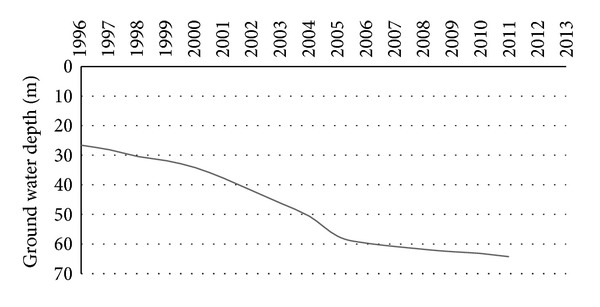
Groundwater depletion in Dhaka City [1].

Dhaka is a densely populated city with an area of 1425 km^2^ [1] which is already labelled as a mega city [2–4]. This significant population craves a larger amount of water for different purposes. Therefore, there is always a shortcoming of supplied water due to an imbalance between demand and supply. Dhaka Water Supply and Sewerage Authority (DWASA) is the only authoritative organization available to deliver consumable water to Dhaka City dwellers. DWASA [1] provides 75% of total demand of water in which about 87% is accumulated from groundwater sources, and the remaining 13% is collected from different treatment plants. Dhaka presently relies heavily on groundwater, with approximately 80 to 90% of demand coming from this source. Overreliance on groundwater sources is depressing the water level. Every year the groundwater table is dropping down around 1 to 3 m due to the extreme amount of withdrawal.  Figure 1 shows the groundwater level depletion trend for Dhaka City. Moreover, scientific studies on the groundwater revealed that excessive exploitation has been lowering the aquifer level, thus limiting natural recharge [5, 6]. Additionally, overexploitation for longer periods may account for several natural hazards such as unexpected landslides, sustained water logging, reduction in soil moisture, and changes in natural vegetation [2, 7–9].

Conjunctive use of groundwater and surface water would be one potential solution to reduce heavy reliance on groundwater. Surface water treatment plants are treating polluted water before delivering it to a supply pipeline. But the level of pollution of surface water has limited the applicability of the treatment process. DWASA supplies 2092.69 million liters of water daily against the current demand for 2815.7 million liters [1], which indicates that the city is facing a huge shortage of water daily. All the scenarios between water demand and supply prevail the immediate need for adopting alternative solutions to release the pressure on water sources. Moreover, current water practices have limited attention to the climate change impacts on water availability [10]. Surveys on climate projections provide evidence on critical impacts of climate on natural water sources that eventually affect human societies and ecosystems [11].

Rainwater harvesting (RWH) could be the most sustainable solution to be included in the urban water management system. It could mitigate the water crisis problem, reduce the burden on traditional water sources, alleviate nonpoint source pollutant loads, control water logging problems, prevent flooding, help in controlling climate change impacts, contribute to the storm water management, and so forth [12–16]. Water scarcity and the limited capacity of conventional sources in urban areas promote RWH as an easily accessible source [17]. The system could be utilized locally and commercially for securing water demand in water-scarce areas all around the world. Harvested rainwater could be idealized and used like supply water if the water-quality parameters satisfy the desired level. The monitoring of collected rainwater is of great concern as it is the potential for health risk because of the presence of chemical and microbiological contaminants [18]. Therefore quality assessment of collected water is essential before use. This paper is mainly focused on scrutinizing and assessing water-quality parameters as per allowable limit and also on the financial benefit acquired by using this technique. Finally this paper suggests a rainwater harvesting system as a potential source of water supply in Dhaka City.

## 2. Water Scenario in Dhaka City

**Figure 2:**
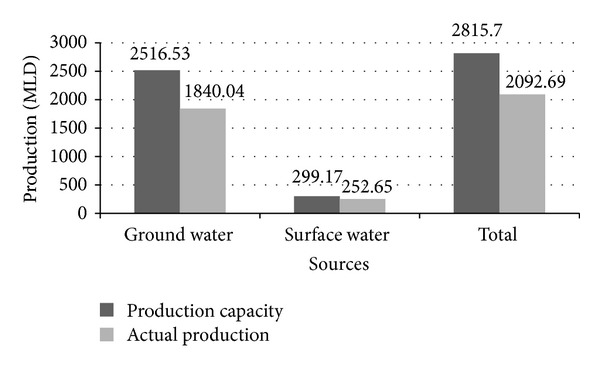
Water production per day in Dhaka city [1].

About 75% of total demand of water in Dhaka is supplied by DWASA, and the rest comes from privately owned tube wells. At present DWASA can yield about 2092.69 million liters (ML) [1] per day in which about 1840.04 MLD is collected from 586 deep tube wells (DTW), and the remaining 252.65 MLD is supplied by two surface water treatment plants [1]. More details are given in Figure 2.

Buriganga, Balu, Turag, and Tongi Khal are the main four water bodies surrounding the city and could be an ideal sources of water supply [19, 20]. But these water bodies already lost their potentiality as sources of supply due to the huge pollutions. Untreated municipal and industrial wastes make the river water so contaminated that most of the water quality parameters surpassed their allowable level. However, the water supply authority mainly relies on groundwater sources and needs to install more tube wells to fulfill demand [21, 22]. Installation of more tube wells must lower the groundwater level. Therefore it is urgent to find a sustainable solution that could alter the usage of groundwater. Rainwater harvesting would be one of the most conceivable and viable solutions to release the pressure on the groundwater table as the system utilizes natural rainwater without affecting groundwater sources.

## 3. Water Supply and Demand Variation

**Figure 3:**
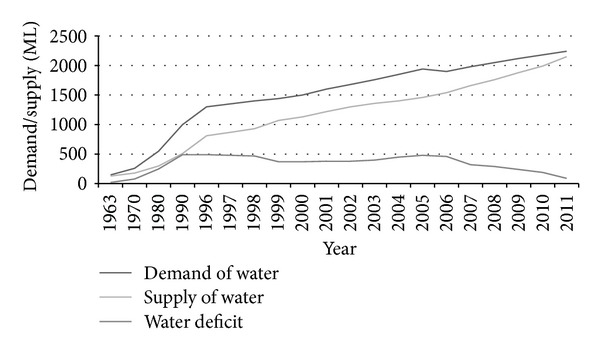
Relation among water demand, supply, and deficit in Dhaka City [1].

In order to understand the variation between demand and supply, the total demand needs to be known. That could be calculated through population data and per capita demand. According to Bro [23], per capita demand for 2006 was about 200 liters, including 10% provisions for commercial use and 40% due to system loss during supply. As per capita demand will be assumed to be decreased in the future by proper inspection and management, for 2015 the total per capita demand will stand at 180 liters per day and for 2025 and 2030 at 160 liters per day. According to DWASA, 2011 [24], the water supply is about 1356.67 MLD (considering service flow with 40% leakages), and the total demand is 2200 MLD (assuming 85% service area). So the deficit is about 843.33 MLD. As demand is more than just supplied water, deficit prevails, which is increasing every day. Therefore the water crisis becomes a normal issue due to this huge deficit in Dhaka City during the dry period. The trend of deficit is due to difference in demand and supply as shown in Figure 3. In 1963 the total demand was 150 million liters (ML), which turned into 2240 million liters in 2011 due to the augmentation of the population. Within 48 years demand became 15 times more than expected. In a similar way, the deficit also crosses predicted values. In 1963 the deficit was 20 ML, and in 2010 it became 190 ML, which was more than calculated. But after that, the shortage became something better than in the previous year. This indicates that supply capacity is improving, and authorities are trying to reduce the shortages. The overall deficiency of supplied water triggers the need for augmentation and improvement of the water supply system to meet the increased demand in future [5].

**Figure 4:**
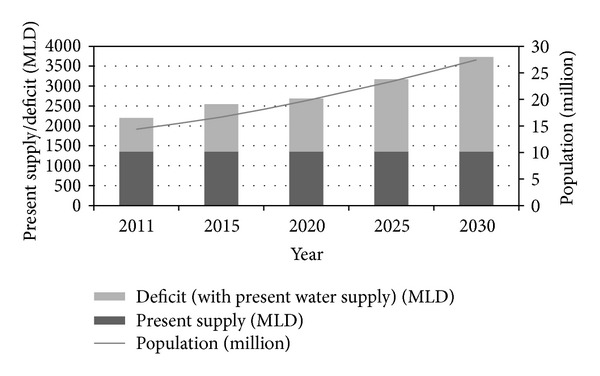
Present water supply, shortage, and population variation for projected years.

Figure 4 shows the variation of the water deficit with the present supply and variation of the population for the projected years. If the present supply prevails for the coming years, the deficit of water will be increasing to a high amount that could not be alleviated within the allowable limit.

**Figure 5:**
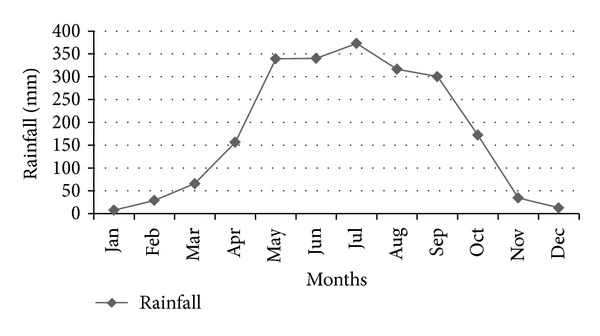
Monthly average rainfall in mm in Dhaka City.

**Figure 6:**
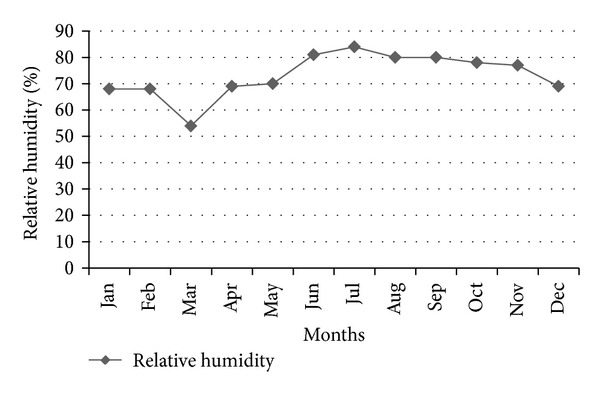
Monthly average relative humidity (%) in Dhaka City.

**Figure 7:**
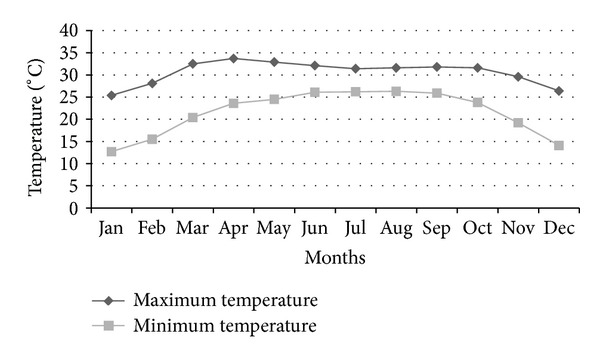
Maximum and minimum temperature (°C) trend in Dhaka City.

Dhaka is located in a hot and humid country, and its annual temperature (25°C) categorizes the city as monsoon climate zone. The city is blessed by a huge amount of rainfall during the monsoon period, which poses ample opportunity to use this rainwater in a sustainable manner [25]. Figures 5, 6, and 7 show the monthly rainfall pattern, monthly average relative humidity, and the maximum and the minimum monthly temperature trend, respectively, for Dhaka City.

The common practices of recharging natural aquifers are by direct rainfall, river water, and direct infiltration and percolation during floods [26]. Overpopulation makes these options inappropriate by reducing the recharge area. Covering the vertical recharge inlets with pavement materials or other construction materials can cause water logging for even small duration heavy rainfall in most areas of Dhaka City. Inadequate storm water management infrastructures and improper maintenance of storm sewer systems further aggravates the scale of this problem. Harvesting of this storm water in a systematic way thus prevents water logging. Furthermore, utilization of collected rainwater highly releases the dependency on groundwater sources.

## 4. Rainwater Harvesting

**Figure 8:**
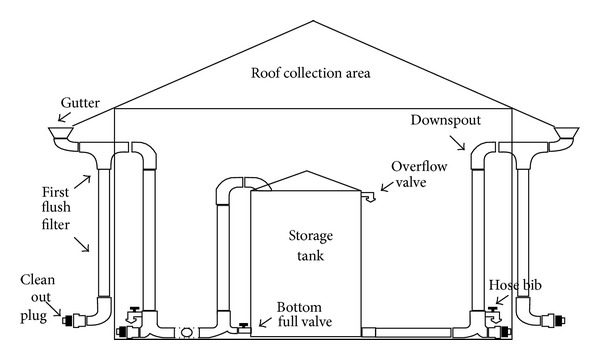
Schematic of a rainwater harvesting system.

Rainwater harvesting is a multipurpose way of supplying usable water to consumers during a crisis period, recharging the groundwater and finally reducing the runoff and water logging during the season of heavy rainfall. Traditional knowledge, skills, and materials can be used for this system. During the rainy season, an individual can collect water on his rooftop and manage it on his own. Reserved rainwater on rooftops can be used for self-purposes or domestic use. Water from different rooftops of a lane can also be collected through a piped network and stored for some time. This water can be then channeled to deep wells to recharge groundwater directly, to ponds to replenish groundwater slowly, and to reservoirs to dilute reclaimed water for nonpotable use.  Figure 8 shows the schematic view of a rainwater harvesting system.

Unless it comes into contact with a surface or collection system, the quality of rainwater meets Environmental Protection Agency standards [27], and the independent characteristic of its harvesting system has made it suitable for scattered settlement and individual operation. If needed, a chemical treatment such as chlorination can be used to purify the water. The acceptance of rainwater harvesting will expand rapidly if methods are treated such as building services and if designed into the structure instead of being retrofitted [28].

## 5. Benefits of Rainwater Harvesting

Rainwater harvesting is a simple and primary technique of collecting water from natural rainfall. At the time of a water crisis, it would be the most easily adaptable method of mitigating water scarcity. The system is applicable for both critical and normal situations. It is an environmentally friendly technique that includes efficient collection and storage that greatly helps local people. The associated advantages of rainwater harvesting are that

- it can curtail the burden on the public water supply, which is the main source of city water;
- it can be used in case of an emergency (i.e., fire);
- it is solely cost effective as installation cost is low, and it can reduce expense that one has to pay for water bills;
- it extends soil moisture levels for development of vegetation;
- groundwater level is highly recharged during rainfall.

## 6. Quality of Rainwater

The quality of harvested rainwater is an important issue, as it could be utilized for drinking purposes. Quality of captured water from roof top depends on both roof top quality and surrounding environmental conditions, that is, local climate, atmospheric pollution, and so forth [11]. Tests must be performed to check its viability and applicability before using as drinking water. Previous researches [29–31] showed that water quality of collected water did not always meet standard limits due to unprotected collection. Local treatment of harvested water could easily make water potable. Again rainwater could be also identified as non-potable sources for the purpose of washing, toilet flushing, gardening, and so forth, where quality is not a great concern. In this respect, treatment of collected water is of no such importance; rather it is used for household purposes. In this paper an assessment has been made on the quality of rainwater collected through a well-maintained catchment system.

## 7. Methodology

Rainwater harvesting is a more effective technology that could be easily undertaken through normal equipment during a water crisis. Qualitative assessment is important before introducing collected rainwater as potable water. In this paper, a case study has been made to check rainwater quality to identify its acceptability and suitability as household water. Water samples were collected from the selected residential building where a rainwater harvesting system was introduced successfully using laboratory prepared plastic bottles to collect samples. The samples were bottled carefully, so that no air bubble is entrained in the bottle. All parameters were measured in the environment laboratory of Bangladesh University of Engineering Technology (BUET).

The maximum amount of rainwater that could be encountered from a roof top is

(1) $$\begin{array}{c} V=A\times R\times C, \end{array}$$

where *V* is the amount of harvestable water, *A* is catchment area, *R* is total amount of rainfall, and *C* is the runoff coefficient.

Equation (1) was used to calculate the amount of harvested water from a residential building located at Dhaka, Bangladesh. The system was designed for meeting water requirements of 60 persons living in the entire building. Total area was about 3600 sq. ft. (square feet). Maximum ground coverage would be around 2250 sq. ft. (considering the floor area rule of RAJUK, the city development authority), and within this area 1850 sq. ft was used as catchment area where rainwater was collected. Per capita water consumption is about 135 lpcd for conservative use. The total demand for this building stands at about 8100 liter per day and 243,000 liters per month. In a practical case, the size of the catchment area is taken from maximum ground coverage. To get an overview of the amount of collected rainwater, monthly average rainfall data from January to December has been considered, including the dry and monsoon periods. The runoff coefficient value was taken as 0.85. For analysis purpose, a one-year rainfall data were considered. Volume of collected rainwater was also an important aspect in introducing rainwater for domestic purposes. In the selected time frame, maximum volume of water was collected during June, 2012, which was about 4.5 m^3^ and a minimum was collected during October, 2011. Significant amount of water could be collected during heavy rainfall. From this point of view, it could be said that, with larger catchment area, amount of harvested water would be significant to be used in household works.

## 8. Results and Discussion

The main focus of this paper relies on several aspects, such as examining the quality of water with respect to standard values, analyzing associated financial benefits in terms of cost, and considering water and energy conservation and lastly suggesting the system as a potential source of water both in normal and critical situations.

**Figure 9:**
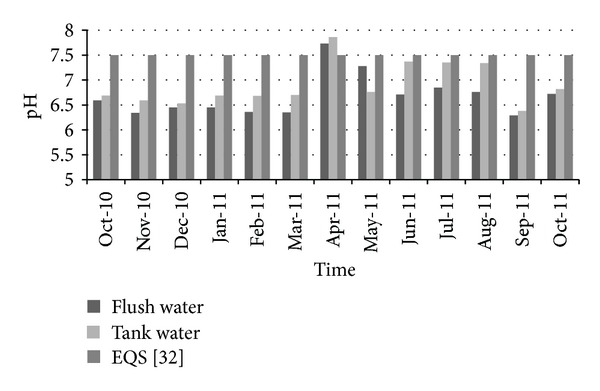
Variation of pH over time.

In this section, the quality of harvestable water was checked considering several parameters such as pH, fecal coliform, total coliform, total dissolved solids, turbidity, NH_3_–N, lead, and BOD_5_. The time period for analysis was from October 2010 to October 2011. Two different collecting points were considered: water collected before entering into the storage tank (called first flush water) and water collected from the storage tank (tank water).  Figure 9 shows the variation of pH over time. According to Bangladesh standards for drinking water [32], the allowable limit for pH is 6.5 to 8.5. Results showed that pH value for both flash and tank water was very near to this range during the tested time period. Therefore, the pH level of collected water did not pose any threat to water quality and conformed to the standard limit.

**Figure 10:**
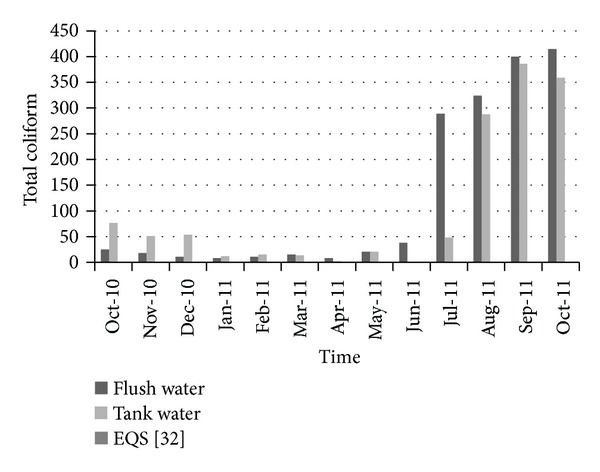
Variation of total coliform over time.

**Figure 11:**
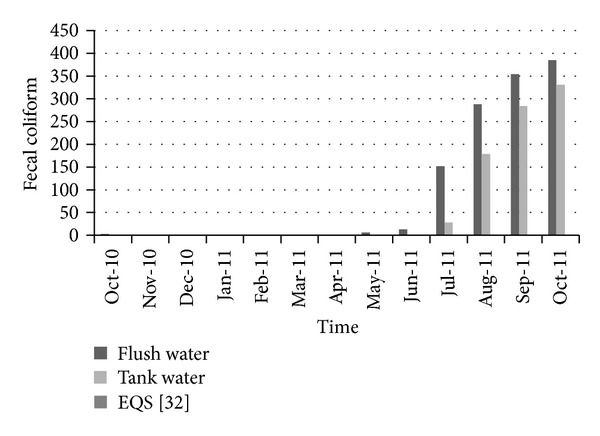
Variation on fecal coliform with time.

Figure 10 shows the variation of total coliform over time. The number of total coliforms present in the water was quite low until June 2011. After that a large number of total coliform grew in both flash and tank water.  Figure 11 shows the variation of fecal coliform over time. In the case of drinking water, it is expected that water should be free from all types of fecal and total coliforms. In the present case, at first in October 2010, few fecal coliforms were found in water. It remains zero until March 2011. But after that there was an increasing trend in the number of fecal coliform. In October 2011, there was huge number of fecal coliform, which is not expectable for drinking water. In both cases (fecal and total coliform), at first when rainwater was harvested, growth of coliform was lower but with time those increased to a large quantity. From June 2011, rainfall was not adequate and maintenance was not proper, which is why coliform grew to a huge quantity in the stored unused water. As pure water should be free from all kinds of coliforms, proper maintenance of tank and catchment areas could minimize coliform level and make rainwater safe for household purposes.

**Figure 12:**
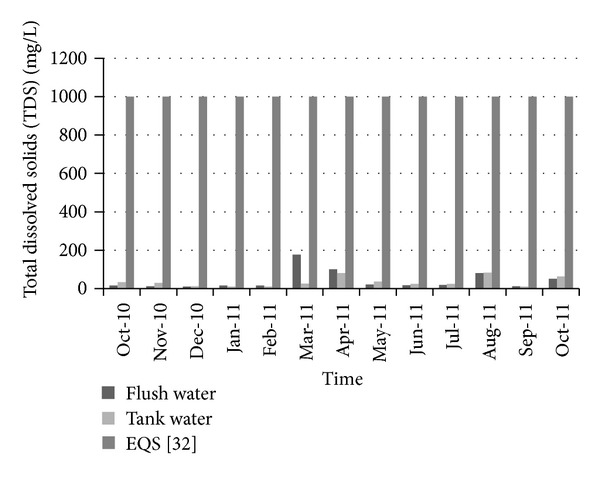
Variation of total dissolved solids over time.

**Figure 13:**
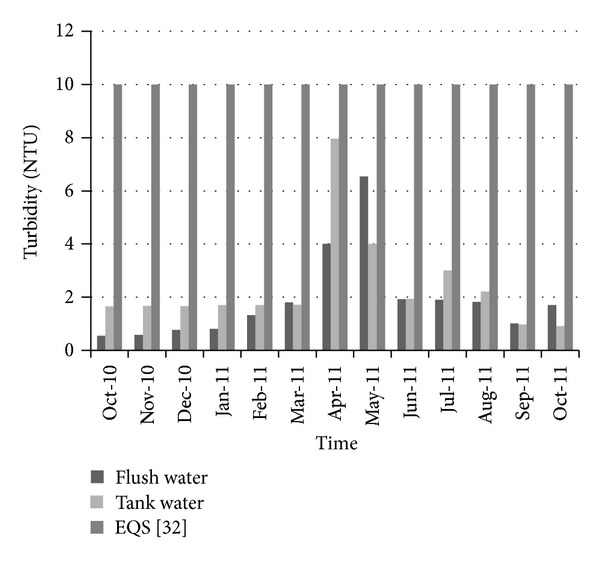
Variation of turbidity over time.

**Figure 14:**
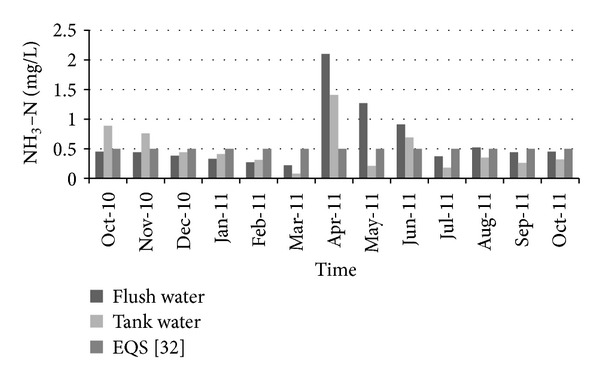
Variation of NH_3_–N over time.

Figure 12 shows the variation of total dissolved solids over time. The allowable limit for total dissolved solids (TDS) in drinking water is about 1000 (mg/L) according to Bangladesh standards for drinking water [32]. For all the selected periods, the total dissolved solids in collected water were quite lower than the standard limit. Therefore total dissolved solids did not pose any threat to water used for drinking purposes.  Figure 13 shows the variation of turbidity over time. The standard limit for turbidity is 10 NTU. The measured turbidity level in collected water was below this standard limit. Therefore rainwater could be considered satisfactory from an aesthetic point of view. In a similar way, the NH_3_–N level was quite below the standard limit (0.5 mg/L) during the collection period (Figure 14).

**Figure 15:**
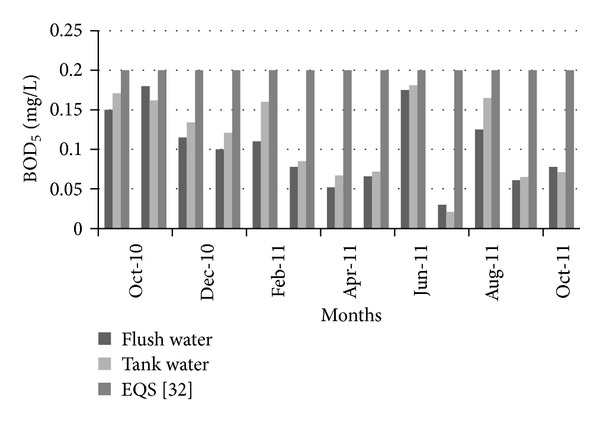
Variation of BOD_5_ over time.

**Figure 16:**
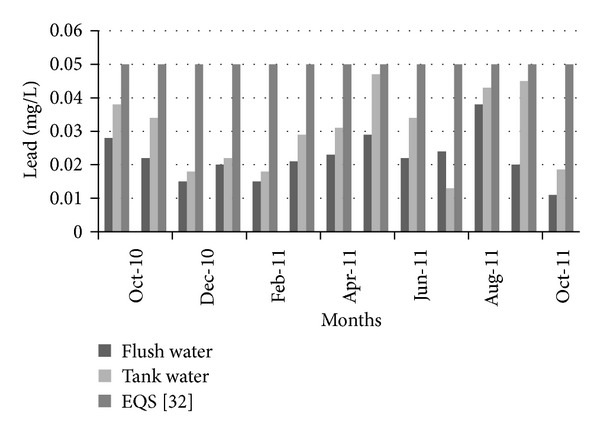
Variation of lead over time.

Figure 15 shows the variation of BOD_5_ in the collected flash and tank water. In all of the selected time period, BOD_5_ is less than the Bangladesh standard for drinking water [32]. Another thing, BOD_5_ became less in flash water than in tank water. Due to the lack of proper maintenance, BOD_5_ increased in the tank water. Further treatment may make water more usable for household work. In order to analyze the water quality in terms of lead concentration in collected water, tests were performed, which found that lead concentration always remained below the allowable limit according to the Bangladesh standards for drinking water [32].  Figure 16 shows the variations of lead concentrations with time.

## 9. Cost Effectiveness Analysis

**Figure 17:**
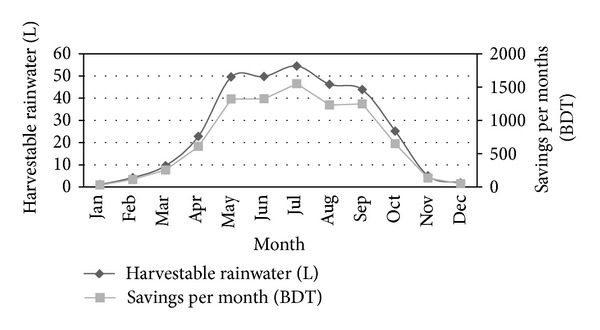
Month-wise harvestable amount of rainwater and the associated cost savings.

Thefinancial benefit associated with a rainwater harvesting system is solely connected with cost. The associated costs of a rainwater harvesting system are for installation, operation, and maintenance. Of the costs for installation, the storage tank represents the largest investment, which can vary between 30% and 45% of the total cost of the system dependent on system size. A pump, pressure controller, and fittings in addition to the plumber's labor represent other major costs of the investment. A practical survey showed that (in Dhaka) the total cost related to construction and yearly maintenance of a rainwater harvesting system for 20 years' economic life is about 30000 BDT. This cost includes construction cost of tanks, gutters, and flushing devices and labor cost [33]. In the present case study, about 313.80 thousands liter water can be harvested from rain over one year. This amount of water could be collected within 1850 sq. ft catchment area and considering monthly rainfall data. The yearly consumption of this selected building stands at 2916 thousands liters. Therefore utilizing harvested rainwater for this building can save up to 11% of the public water supply annually. This volume of rainwater can serve a building with 60 members for about 1.5 months in a year without the help of traditional water supply.  Figure 17 shows the month-wise harvestable amount of rainwater and the associated amount of cost savings. Furthermore, considering DWASA current water bill, about 8359.70 BDT can be saved per year, and about 125395.30 BDT can be saved in 15 years if rainwater is used for daily consumption. So, within three to four years, the installation cost of a rainwater harvesting system can be easily returned. Moreover, the building owner would be exempted from paying large amount of water bill as well as additional taxes and fees charged by the city authority with the water bill if rainwater is utilized for daily consumption. Cost comparison and associated benefit between a rainwater harvesting system and traditional water supply system encountered and revealed a rainwater harvesting system as a cost-effective technology.

## 10. Water Savings Strategy

Rainwater harvesting system plays an important role in developing sustainable urban future [24]. Availability of water of serviceable quality from conservative sources is becoming limited day by day due to huge demand. Rainwater provides sufficient quantity of water with small cost. Hence, the system can promote significant water saving in residential buildings in many countries. Herrmann and Schmida [35] studied that potential saving of roof captures water was about 30–60% of potable water demand in a house depending on the demand and catchment area. Coombes et al. [36] analyzed 27 houses in Australia with rainwater harvesting system and found that about 60% of potable water could be saved. Ghisi et al. [37] performed investigation on collected rainwater in Brazil and found that about 12–79% of potable water could be saved depending on the size of roof tank. Most of the researches on rainwater harvesting systems (RWHS) revealed that water conservation achieved through RWHS is quite significant especially in places where water is not easily available to consumers.

## 11. Energy and Climate

Conventional use of water imparts critical impacts on natural resources. Water collection from ground and surface sources, treatment, and distribution are closely associated with energy consumption, however, being related to climate consequences. The extraction of water from the sources, the treatment of raw water up to the drinking standards and the delivery of water to the consumers require high energy. Moreover, there should be some energy losses during performing extracting, treating, and delivering of water. Therefore, the water sector consumes a huge amount of electricity from local and national grid. Approximately 300 billion kilowatt hours of energy could be saved if potable water demand could be reduced by 10% [38]. Adoption of RWHS is one of the most potential solutions that could save energy directly by reducing potable water demand.  Table 1 represents the estimated energy required to deliver potable water to consumers. Reduction of water demand by 1 million gallons can result in savings of electricity use by 1,500 kWh. In the present case study, with an 1850 sq. ft. catchment area, about 69,026 gallons (313.8 thousands liters) could be harvested over one year. However, this amount could reduce potable water demand and approximately 100 kWh electricity could be saved in the selected residential building by introducing rainwater capturing system. Integrating rainwater harvesting system with the conventional water collection and distribution approach in residential as well as large scale, nonresidential applications suggest a potential method of reducing energy use. However, limiting energy demand has critical impact on carbon dioxide emissions, as release of carbon dioxide is closely associated with electricity generation. There should have sufficient reduction in carbon dioxide emissions when fossil fuel is used for power generation. Hence, limited contribution is to be expected from lower carbon release in climate change concept.  Table 2 showed the carbon dioxide emissions from electric power generation.

However, water use should be critically judged from availability, safety, and sustainability of natural resources. Energy conservation is a critical component in sustainability concern. Decreased use of conventional potable water reduces energy demand that in turn reduces emission of carbon dioxide. Integrated water management approach with rainwater harvesting along with gray water and reclaimed water reuse could limit contributions to climate change and conserve limited water and energy resources.

## 12. Future Action Plan

Rainwater is one of the advantageous methods of using natural water in a sustainable manner. Rain is a blessing of nature. Densely populated cities with a water crisis and adequate rainfall should adopt this technology. Cities like Dhaka, where water is a major concern during dry periods, should introduce this system along with its traditional water supply system. Pressure on groundwater tables thus could be prevented, and natural recharging would also be proceeded through this system. Regular maintenance of harvested water might make it suitable for daily consumption. Water shortages will become the most concerned issue all around the world in the future. Therefore city planners should rethink of the possibilities, outcome, and benefits of a rainwater harvesting system and should create policies to make the system easily available to everyone. The following research could be made in future.

- This study focused only on rainwater harvesting system on a small scale basis. Further research could be performed on large scale residential, commercial or industrial sector.
- Comparisons could be made with rainwater harvesting systems to conventional ground water system on the basis of quality, quantity, environmental impacts, energy saving, water conservation, economy, and so forth.
- Case studies could be investigated to evaluate energy consumption in rainwater system with ground water system in a large scale. In a more applied setting, energy efficiencies of large scale rainwater harvesting systems should be analyzed to help determine the future of rainwater harvesting as a valuable technology for providing water, a crucial resource that is becoming more depleted with the ever increasing population and water demand.
- A comprehensive cost-benefit analysis should be performed on different climate regions to get essential insight on the economic viability of rainwater harvesting system (RWHS).
- More detailed and advanced research on impacts on climate factors, human health risk, and potential ecological aspects should be performed in a large scale.
- More comprehensive studies for better quantification of energy and climate factors should be made for proper development of the system.
- Rainwater could be highly polluted by pesticides in any agricultural region. Hence, biological and chemical analysis should be done before adopting harvested rainwater as a source of daily water.

## 13. Conclusion

Water shortage is one of the critical problems in Dhaka City. This problem is not new one, and it cannot be solved overnight. As DWASA relies on groundwater abstraction through deep tube wells to overcome the excessive demand, the water table is lowering day by day, and the recharge of groundwater table is facing difficulties. Rainwater harvesting is an effective option not only to recharge the groundwater aquifer but also to provide adequate storage of water for future use. This paper tried to focus on the sustainability and effectiveness of a rainwater harvesting system in terms of quality. Water was collected in a well maintained catchment system from rain events over one year and chemical analysis was performed regularly to observe the quality of collected water. The overall quality of rainwater was quite satisfactory and implies that the system could be sustained during critical periods as well as normal periods. Additionally, the system is cost effective as large amounts of money can be saved per year. Energy conservation and related reduced emissions are crucial parts of this system. Moreover, increased awareness on water crisis has led rainwater harvesting to be proposed as a community facility. The small and medium residential and commercial construction can adopt this system as sustainable option of providing water. It is almost the only way to upgrade one's household water supply without waiting for the development of community system. The system could become a good alternative source of water supply in Dhaka City to cope up with the ever-increasing demand and should be accepted and utilized by the respective authorities as well as by the city dwellers.

## Figures and Tables

**Table 1:** Energy consumption in conventional water resources system [34].

Activity	Energy consumption (kWh/MG)
Supply and conveyance	150
Water treatment	100
Distribution	1,200

Total	1,450

**Table 2:** Carbon dioxide emission from water treatment and distribution system [39].

Fuel type	CO_2_ output rate pounds (CO_2_/kWh)	Drinking water energy demand (kWh/MG)	CO_2_ output rate per MG water delivered (CO_2_/kWh)
Coal	2.117		2,906
Petroleum	1.915	1,406	2,680
Natural gas	1.314		1,840
